# Characterization of the linkage disequilibrium structure and identification of tagging-SNPs in five DNA repair genes

**DOI:** 10.1186/1471-2407-5-99

**Published:** 2005-08-09

**Authors:** Kristina Allen-Brady, Nicola J Camp

**Affiliations:** 1 Genetic Epidemiology, Department of Medical Informatics; University of Utah School of Medicine; 391 Chipeta Way, Suite D; Salt Lake City, Utah, 84108, USA

## Abstract

**Background:**

Characterization of the linkage disequilibrium (LD) structure of candidate genes is the basis for an effective association study of complex diseases such as cancer. In this study, we report the LD and haplotype architecture and tagging-single nucleotide polymorphisms (tSNPs) for five DNA repair genes: ATM, MRE11A, XRCC4, NBS1 and RAD50.

**Methods:**

The genes ATM, MRE11A, and XRCC4 were characterized using a panel of 94 unrelated female subjects (47 breast cancer cases, 47 controls) obtained from high-risk breast cancer families. A similar LD structure and tSNP analysis was performed for NBS1 and RAD50, using publicly available genotyping data. We studied a total of 61 SNPs at an average marker density of 10 kb. Using a matrix decomposition algorithm, based on principal component analysis, we captured >90% of the intragenetic variation for each gene.

**Results:**

Our results revealed that three of the five genes did not conform to a haplotype block structure (MRE11A, RAD50 and XRCC4). Instead, the data fit a more flexible LD group paradigm, where SNPs in high LD are not required to be contiguous. Traditional haplotype blocks assume recombination is the only dynamic at work. For ATM, MRE11A and XRCC4 we repeated the analysis in cases and controls separately to determine whether LD structure was consistent across breast cancer cases and controls. No substantial difference in LD structures was found.

**Conclusion:**

This study suggests that appropriate SNP selection for an association study involving candidate genes should allow for both mutation and recombination, which shape the population-level genomic structure. Furthermore, LD structure characterization in either breast cancer cases or controls appears to be sufficient for future cancer studies utilizing these genes.

## Background

Candidate gene association studies are a powerful study design for complex diseases such as cancer. Advances in association studies have been furthered by the recent discovery of single nucleotide polymorphisms (SNPs); their vast density throughout the genome, ease of genotyping and moderate cost contribute greatly to their utility. Association testing is efficient when the SNPs being analyzed represent the entire genetic variation of the gene. It has been suggested that nearby SNPs are organized into regions of high linkage disequilibrium (LD) separated by short segments of very low LD [1-6]. In Caucasians, high LD regions may vary in length from a few kb to >300 kb[2,6,7]. Regions of high LD contain redundant information and can be reduced to smaller subsets of tagging-SNPs (tSNPs)[8], such that tSNPs identify all common haplotypes within the region of high LD. A number of algorithms have been proposed to define regions of high LD and tSNPs[4,8-14]. Thus far, no consensus of which algorithm is best has been achieved. Several studies have suggested the utility of matrix decomposition algorithms.[12,13,15-17]. One advantage of these algorithms is that SNPs in high LD are not required to be contiguous nor mutually exclusive, a flexibility that is necessary for analyzing small genomic regions and rare variants. Further, these methods are stable with regards to marker density, minor allele frequency, analysis window, and possible analysis window length[18].

Growing evidence appears to suggest that tumorigenesis is a multi-step process of genetic alterations that transform a normal human cell into a malignant derivative[19]. The ability of a cell to maintain genomic stability through DNA repair mechanisms is essential to prevent tumor initiation and progression. A number of different types of cancer have been attributed to defective DNA repair including xeroderma pigmentosum[20], hereditary nonpolyposis colorectal cancer[21], and breast cancer due to mutations in *BRCA1 *and *BRCA2 *as well as other DNA repair genes (e.g., *ATM*, *TP53 *and *CHK2*)[22]. Many published candidate gene association studies involving DNA repair genes and cancer risk have assessed risk by examining a single SNP per gene or a single locus at a time analysis approach. Unfortunately, the former approach is often inadequate in comprehensively accounting for the genetic variation of a gene, and the latter incurs multiple testing corrections, which usually eliminate all or most of the association evidence found. It has been suggested that use of haplotypes in association studies may have increased power over single-allele studies[8]. Descriptions of haplotype diversity and LD structure as well as identification of potential tSNPs will be key for success in candidate gene association studies.

Here we describe haplotypes, LD structure and potential tSNPs in five DNA repair breast cancer susceptibility genes: *ATM*, *MRE11A*, *NBS1*, *RAD50*, and *XRCC4*. We used a matrix decomposition algorithm based on a method of principal components analysis[13]; this method does not require SNPs to be in contiguous block structure. Characterization of the LD structure and tSNPs are necessary for the design of future effective association studies.

## Methods

### Subjects

This study is part of a larger study involving 139 high-risk Caucasian breast cancer families, defined as high risk because cancer rates in these families were significantly higher than the general population rate determined using the Utah Population Database (UPDB) [23-25]. All breast cancer cases in the larger cohort met at least one of the following criteria: 1.) their family tested negative for a *BRCA1 *or *BRCA2 *mutation, 2.) the case themselves tested negative for the same *BRCA1/2 *mutation that was present in their family, or 3.) their family had a low probability of carrying a *BRCA1/2 *mutation based on the number of breast cancer cases present in the family and/or ages at diagnosis of breast cancer within the family. Therefore, all breast cancer cases in the larger study had a low residual probability of their cancer being due to mutations in *BRCA1/2*. Breast cancer diagnosis information was obtained from medical records for the subject or the Utah Cancer Registry.

For this LD characterization study, we selected a panel of 94 individuals (47 female breast cancer cases and 47 female controls), chosen randomly from separate kindreds to ensure independence. Both cases and controls were chosen such that comparisons of LD structure could be made between the groups. The sample size of 188 chromosomes is larger than generally used for this type of study [26-29], but inadequate for an association analysis. This current study is not a case-control study and associations with disease were not assessed.

Blood samples were collected on all subjects and all individuals signed consent to participate this study. This study was approved by the University of Utah Institutional Review Board.

### Genes and SNP selection

For each gene of interest (i.e., *ATM*, *MRE11A*, *NBS1*, *RAD50 *and *XRCC4*), all SNPs available from Applied Biosystems[30], within each gene and the flanking 10 kb on either side, that had been validated to have a minor allele frequency greater than 0.01 in Caucasians were selected. For *ATM *(on chromosome 11q22-q23), which spans approximately 143 kb and contains 64 exons, 14 SNPs were studied with a SNP resolution of 1 SNP/10,489 bp. For *MRE11*A (11q21), which spans approximately 76 kb and contains 20 exons, 11 SNPs were studied with a SNP resolution of 1 SNP/8539 bp. For *NBS1 *(8q21), which contains 16 exons and spans about 51 kb, 5 SNPs were studied with a SNP resolution of 1 SNP/8256 bp. The *RAD50 *gene (5q31) spans approximately 87 kb contains 25 exons, and we studied 10 SNPs at a resolution of 1 SNP/10,533 bp. Finally, for *XRCC4 *(5q13-q14) with 8 exons and approximately 276 kb in length, we studied 21 SNPs at a resolution of 1 SNP/13,198 bp. The vast majority of the SNPs studied were intronic (see Table 1).

### Genotyping

For the *ATM *and *XRCC4 *all SNPs that met the above criteria were genotyped on our panel of 94 subjects. For *MRE11A*, one SNP repeatedly failed to amplify (rs10831224) and was removed from the study.

Genomic DNA was isolated and purified using standard phenol/chloroform DNA extraction. SNP genotyping was performed using the fluorogenic 5' nuclease TaqMan Assay[31] (Applied Biosystems). The TaqMan Assay requires TaqMan PCR Master Mix (Applied Biosystems), which we used according to manufacturer's instructions, yielding a final volume of 5 μl per well. PCR amplification was also performed according to the Applied Biosystems protocol. The 7900HT Sequence Detection System (Applied Biosystems) was used to measure each fluorescent dye-labeled probe specific for each allele studied and results were analyzed with the Sequence Detection Software (Applied Biosystems).

### Haplotype structure and tSNP selection

Haplotypes and haplotype frequencies were estimated from unphased genotype data using an expectation-maximization algorithm, SNPHAP[32]. SNPHAP uses a maximum-likelihood program to predict multilocus haplotypes. Haplotypes with a frequency of at least 0.01 were analyzed using a two-step PCA method[13]. This method does not require that groups of SNPs be contiguous along a DNA fragment and also allows SNPs to be present in more than one group. In step I, LD groups are determined. In brief, the PCA method extracts factors (LD groups) to capture ≥ 90% of the genetic diversity. An LD group is defined as those SNPs that load onto the same factor. In step II, tSNPs are selected for each LD group. Each LD group is considered separately and the PCA method again extracts factors; tSNPs are chosen as the SNPs with the highest factor loading. When a number of SNPs load equally well on an LD group, these can all be considered potential tSNPs. Under such circumstances, we selected the single SNP that performed best in the genotyping assay. This was done in order to minimize errors in allele calls.

We compared our genotype data for *ATM*, *MRE11A*, and *XRCC4 *with genotyping data for these same genes obtained from Applied Biosystems (ABI)[30] on 45 Caucasians. We found good concordance in allele frequencies between the data sets. Further, we applied the same LD characterization to both data sets and found excellent concordance in the LD groups and potential tSNPs (see Results). We therefore characterized LD groups and tSNPs for *NBS1 *and *RAD50 *using the genotyping data available online.

We also examined whether differences existed between LD group structure and tSNP selection when cases and controls were considered separately. This analysis could only be performed for *ATM*, *MRE11A*, and *XRCC4*.

## Results

Characteristics of the SNPs studied are listed in Table 1. Minor allele frequencies from our 94 subjects compared well with those listed by Applied Biosystems[30]. Despite the very low minor allele frequencies in some of the SNPs studied, we observed heterozygosity for all SNPs genotyped.

Table 2 lists the haplotypes with a frequency > 0.01 obtained from SNPHAP, and the LD group designation and the tSNPs that were selected using the PCA method, for *ATM*, *MRE11A*, and *XRCC4*. Haplotypes are reported using the standard convention of designating the major allele as '1' and the minor allele as '2', in order to more easily spot occurrences of the minor allele. Please see Table 1 for the corresponding base pair change. For *ATM*, 7 haplotypes overall were observed and 5 had a frequency > 0.01. Using the PCA method, a single LD group was identified, encompassing the entire gene and accounting for 98.8% of the genetic variance across the gene. From this single LD group, a single tSNP (A13) was selected.

For *MRE11A*, we observed 9 haplotypes in total and 6 with frequency > 0.01. From the PCA analysis, four LD groups were identified based on these 6 haplotypes with a frequency > 0.01, and accounted for 99.1% of the genetic variance. The LD groups did not conform to haplotype blocks. SNP M4 separated LD group 1 into two parts and M8 separated LD group 2. Each LD group was represented by a single tSNP, such that the tSNP set contained 4 tSNPs (M6, M10, M11, and M14).

For *XRCC4*, we observed 26 haplotypes overall; 13 of which had a frequency >0.01. From the PCA method, four LD groups were observed which accounted for 97.2% of the variance. Similarly to *MRE11A*, the LD groups were not contiguous blocks. LD group 1 was divided by X9 and LD group 2 was divided by X15. Each of the LD groups could be represented by a single SNP resulting in the tSNP set (X2, X9, X14, and X21).

Table 3 shows the LD groups and tSNPs for *ATM*, *MRE11A *and *XRCC4 *using our panel of 94 subjects and using the 45 Caucasian subjects from Applied Biosystems[30]. For these three genes, we observed the same number of LD groups containing precisely the same SNPs for both data sets. The difference between the results was in the number of potential tSNPs for each LD group. For the majority of LD groups, the potential tSNPs using Applied Biosystems data were a subset of those from our data. This is perhaps expected, because our sample size was more than double their size and is therefore capable of better resolution.

Table 4 lists the haplotypes, LD group designation, potential tSNPs, and tSNP selected per group for *NBS1 *and *RAD50 *using the Applied Biosystems' data. For *NBS1*, 6 haplotypes overall were observed and all 6 haplotypes had a frequency > 0.01. Using the PCA method, two LD groups were identified and accounted for 93.8% of the variance. Two tSNPs were sufficient to tag these groups (N1, N2). However, N5 could replace N2 with no reduction in the variance explained. For the *RAD50 *gene, in order to include two available rare SNPs in the analysis, we lowered the haplotype acceptance threshold to 0.009. We observed a total of 14 haplotypes, 10 with a frequency greater than 0.01. Using the PCA method, we identified three LD groups, which accounted for 91.5% of the variance. Similarly to *MRE11A *and *XRCC4*, the LD groups for *RAD50 *were not contiguous blocks. Three tSNPs were sufficient to tag the groups (R1, R3, and R10), although R5 could replace R1 and R6 could replace R3 with no loss of variance explained.

For *ATM*, *MRE11A*, and *XRCC4*, we compared haplotypes and LD structure between the breast cancer cases and controls. For *ATM *and *XRCC4 *no difference in the LD structure was observed when cases and controls were analyzed separately. For the *MRE11A *gene differences in LD structure were noted, however, these were minor and likely attributable to small sample size since the differences were driven by 3 rare haplotypes (frequency = 0.02).

## Discussion

Identification of the most informative markers to use in a large-scale association analysis for studies of complex disease, such as breast cancer, is critical to the success of the study. The key to this process is to select SNPs that are most informative about the underlying haplotype structure in a population of interest. As haplotype based designs have been suggested as being more powerful than the single-allele approach for association studies[8], a haplotype-based approach should result in more accurate and definitive findings. In this study, we have described haplotypes and characterized the LD structure of the *ATM*, *MRE11A*, and *XRCC4 *genes using a panel of 94 subjects, including breast cancer cases from high-risk breast cancer families as well as controls. Further, we identified tSNPs that can be used in future haplotype-based association studies. A similar analysis was performed for *NBS1 *and *RAD50 *using publicly available genotype data. We identified, using Principal Components Analysis[13], a single LD group for *ATM*, four noncontiguous LD groups for *MRE11A*, two LD groups for *NBS1*, three noncontiguous LD groups for *RAD50*, and four noncontiguous LD groups for *XRCC4*. In each case, the LD groups captured greater than 90% of the variance of the total SNPs available from Applied Biosystems across the gene. Furthermore for each gene, we present tSNPs that could be selected to represent the gene.

It is of interest that the LD structure for three of these five DNA repair genes did not conform to the haplotype block model, that is, that the LD groups did not contain contiguous SNPs. This was true whether the genotyping data came from our own study or from Applied Biosystems. Although we did not directly sequence these genes to identify all possible variants, the discontinuity we observed illustrates that the underlying LD structure cannot conform to contiguous haplotype blocks. A more flexible LD group representation (as supported under principle components analysis) fit the data better and appears to be stable to differences in minor allele frequency. Similar findings of a complex pattern of LD structure were recently reported in a high-resolution study of the ELAC2 gene[15]. Our results suggest that when studying small genomic regions and low frequency variants (<0.2), mutation is an important dynamic in LD structure, and the simple recombination-only model used in classical haplotype block methods does not fit the data well and hence will lead to a poor selection of tSNPs.

Due to the stability of the results for *ATM*, *MRE11A *and *XRCC4*, we pursued two additional DNA repair genes of interest (i.e., *NBS1 *and *RAD50*). Applied Biosystems provides freely-available genotyping data for four ethnically diverse populations of 45 subjects in each, therefore, even with limited funds, the haplotype structure and selection of tSNPs can be estimated for a study prior to any genotyping costs. However, caution must be used if this option is exercised as one's population must be one of Applied Biosystems' ethnic cohorts (i.e., Caucasian, African American, Chinese, or Japanese) and our experience is that occasionally errors exist in the data.

Of the genes studied here, only *ATM *has previously been studied in any depth for LD structure. The reason that *ATM *has received so much attention is that patients with the recessive disease ataxia-telangiectasia, due to a mutation in the *ATM *gene, have a 100-fold increased risk of cancer[33,34] and obligate heterozygous carriers of *ATM *mutations may have an increased risk of cancer, particularly breast cancer [35-39], although this finding is controversial[40,41]. Extensive LD across the *ATM *gene has previously been reported [42-44], and sequence analysis reveals that *ATM *polymorphisms are relatively rare resulting in low overall sequence diversity[44]. Thus, it follows that only a small number of haplotypes have been found, particularly in Caucasian populations of European descent. Thorstenson *et al *[44] predicted seven haplotypes in populations throughout the world, only three of which were found in Europeans or the Americans. Bonnen *et al *[43] identified 22 unique haplotypes, seven of which occurred in Caucasians, and only five of these occurred at a frequency of greater than 5% among Caucasians. We observed five haplotypes for the *ATM *gene, but only two of these could be considered common haplotypes (>0.01) and together accounted for 96% of all chromosomes. A recently published study using those haplotypes defined by Thorstenson *et al*[44] and Bonnen *et al*[43] identified five haplotype tagging-SNPs that were necessary to capture all of these haplotypes with a frequency >1%[45]. In our study, which is limited to Applied Biosystems' validated SNPs, we found that one tSNP was sufficient to represent 98.8% of the total genetic variance for all the SNPs available. The results of our study differed from these other studies due most likely to differences in the minor allele frequency range of the SNPs utilized. Our minor allele frequency for the 14 SNPs studied in the *ATM *gene varied minimally from 0.43 – 0.45. Thorstenson *et al*[44] and Bonnen *et al*[43] included 2 and 3 SNPs, respectively, that had minor allele frequencies <0.25. Population structure exists in SNP-allele frequencies[43] and as observed by the results of this study, exclusion of rarer SNPs has an impact on the frequency of haplotypes that are observed.

Comparison of haplotype and LD structure between cases and controls for *ATM*, *MRE11A*, and *XRCC4 *indicated that LD structure for these genes were similar in both groups. Results for *ATM *and *XRCC4 *were identical and only minor differences in LD structure were noted for *MRE11A *due to three rare haplotypes. A recent study has reported that rare haplotypes may be important for disease susceptibility and in their study these rare haplotypes had significant effects on their phenotype of interest[46]. Therefore, if rare haplotypes are of interest to an investigator, it may be prudent to characterize LD in both cases and controls and select tSNPs that comprehensively cover the diversity of both groups. However, most studies to date have empirically found that LD structure is similar across phenotype[1,47]. If major differences in LD structure were to exist, this would have a profound effect on guidelines for tSNP selection and for application of projects such as the HapMap[48,49].

Some limitations are inherent in this study and must be pointed out. First, we did not sequence our genes of interest and thus all of the genetic diversity within these genetic regions may not be captured. Our results must be interpreted in light of this. The gold standard is to identify all variants within a gene and select a subset of tSNPs from this set. It would be interesting to evaluate the robustness of our findings using sequence data. However, the SNPs examined were relatively evenly spaced, on the order of 1 SNP every 10 kb, and our results are important as they illustrate how smaller budget studies can best select tSNPs. Second, our sample size was modest (188 chromosomes), although larger than other previous studies examining LD and tSNPs [26-29]. Finally, haplotype block and haplotype-tagging SNP analyses have been suggested to only be reliable when markers are dense, otherwise marker sets have considerable loss of information[50]. This result may extend to PCA methods, however, the matrix decomposition algorithm used has been suggested to be stable with regards to varying levels of marker density[18].

## Conclusion

In conclusion, we have described haplotypes, linkage disequilibrium structure, and identified tSNPs from all available Applied Biosystems' validated SNPs in *ATM*, *MRE11A*, *NBS1*, *RAD50*, and *XRCC4 *genes in a Caucasian population. As has been found for other genes, we identified LD structures that did not conform to contiguous haplotype block structures. This illustrates the importance of using flexible methods, such as matrix decomposition, that allow for multiple population dynamics such as recombination, mutation and selection. Although the gold standard for SNP characterization across a candidate gene is sequencing to identify all variants, we describe a low-budget means to characterize the LD structure and select tSNPs using publicly available data. Comprehensive characterization of the LD structure at genes of interest will be essential for future, effective association studies.

## Electronic database information

The data from the 94 breast cancer case and control subjects for these tables is publicly available at  under Supplemental Materials to Publication. On request from Dr. Nicola Camp a username and password to access the data will be given.

## Competing interests

The author(s) declare that they have no competing interests.

## Authors' contributions

KAB assisted in the study design, performed the genotyping, and drafted the manuscript. NJC conceived of the study and its design and helped to draft the manuscript. All authors read and approved the final manuscript.

## Pre-publication history

The pre-publication history for this paper can be accessed here:



## Figures and Tables

**Table 1:** Characteristics of SNPs analyzed

Gene	SNP Code	SNP ID	Base change*	Position^†^	MAF^‡^	ABI reported MAF^§^	# bp from the most 5' SNP
ATM	A1	rs228589	T/A	Flanking	0.45	0.33	0
ATM	A2	rs228591	G/A	mRNA-utr	0.45	0.33	4125
ATM	A3	rs641605	T/C	Intron	0.45	0.33	8,711
ATM	A4	rs228599	A/G	Intron	0.44	0.31	14,452
ATM	A5	rs600931	T/C	Intron	0.45	0.35	24,127
ATM	A6	rs228592	A/C	Intron	0.45	0.33	29,981
ATM	A7	rs664677	T/C	Intron	0.43	0.33	49,974
ATM	A8	rs1003623	T/C	Intron	0.45	0.33	59,374
ATM	A9	rs609261	C/T	mRNA-utr, intron	0.45	0.32	64,926
ATM	A10	rs645485	G/A	Intron	0.45	0.32	75,655
ATM	A11	rs673281	A/G	Intron	0.45	0.31	88,861
ATM	A12	rs227061	G/A	mRNA-utr, intron	0.45	0.34	112,121
ATM	A13	rs227062	A/G	mRNA-utr, intron	0.45	0.33	112,175
ATM	A14	rs652311	A/G	Flanking	0.45	0.36	146,861
MRE11	M1	rs646130	T/C	Flanking	0.3	0.39	0
MRE11	M2	rs491404	G/C	Flanking	0.3	0.4	9192
MRE11	M3	rs10831227	G/A	Intron	0.3	0.4	16,336
MRE11	M4	rs601341	G/A	Intron	0.38	0.36	28,536
MRE11	M5	rs554715	T/C	Intron	0.3	0.4	32,986
MRE11	M6	rs556477	A/G	Intron	0.3	0.4	40,565
MRE11	M7	rs1805365	A/G	Intron	0.02	0.02	61,721
MRE11	M8	rs680695	A/G	Intron	0.34	0.36	72,913
MRE11	M9	rs1009455	C/G	Intron	0.02	0.01^||^	85,033
MRE11	M10	rs1009456	C/A	locus-region, mRNA-utr	0.01	0.02	87,401
MRE11	M11	rs10831234	C/T	Flanking	0.09	0.06	93,946
NBS1	N1	rs12680687	G/T	Intron	- **	0.28	0
NBS1	N2	rs709816	A/G	Coding-synon	-	0.45	16,323
NBS1	N3	rs1805790	C/T	Intron	-	0.39	23,313
NBS1	N4	rs741778	C/G	Intron	-	0.36	33,415
NBS1	N5	rs1805841	C/G	Intron	-	0.45	41,282
RAD50	R1	rs2522406	G/A	Flanking	-	0.01	0
RAD50	R2	rs2244012	C/T	Intron	-	0.19	12,116
RAD50	R3	rs2299015	T/G	Intron	-	0.19	12,388
RAD50	R4	rs2299014	G/T	Intron	-	0.41	14,290
RAD50	R5	rs2706377	A/G	Intron	-	0.01	50,388
RAD50	R6	rs2301713	C/T	intron	-	0.19	62,887
RAD50	R7	rs2040703	C/G	Intron	-	0.22	83,149
RAD50	R8	rs2240032	C/T	Intron	-	0.18	88,018
RAD50	R9	rs1800925	C/T	Flanking	-	0.19	103,700
RAD50	R10	rs2066960	C/A	Flanking	-	0.17	105,326
XRCC4	X1	rs1993948	T/A	Flanking	0.46	0.47	0
XRCC4	X2	rs1478485	G/A	mRNA-utr	0.47	0.45	8247
XRCC4	X3	rs11951257	T/C	Intron	0.47	0.45	31,031
XRCC4	X4	rs10045104	C/T	Intron	0.43	0.42	40,082
XRCC4	X5	rs6452526	C/T	Intron	0.47	0.43	64,531
XRCC4	X6	rs1382369	G/A	Intron	0.47	0.43	69,149
XRCC4	X7	rs1382368	C/T	Intron	0.47	0.41	78,795
XRCC4	X8	rs1382363	C/T	Intron	0.47	0.42	80,292
XRCC4	X9	rs13180316	G/A	Intron	0.23	0.26	87,173
XRCC4	X10	rs11741420	A/T	Intron	0.47	0.44	98,452
XRCC4	X11	rs2731861	T/C	Intron	0.47	0.45	112,984
XRCC4	X12	rs2662238	G/A	Intron	0.46	0.45	127,027
XRCC4	X13	rs1039786	C/T	Intron	0.46	0.45	127,761
XRCC4	X14	rs963248	T/C	Intron	0.19	0.16	161,614
XRCC4	X15	rs301276	G/A	Intron	0.23	0.23	175,451
XRCC4	X16	rs35268	T/C	Intron	0.16	0.13	216,216
XRCC4	X17	rs301286	T/C	Intron	0.16	0.18	230,675
XRCC4	X18	rs301289	C/T	Intron	0.17	0.17	233,955
XRCC4	X19	rs2386275	G/A	Intron	0.09	0.12	270,260
XRCC4	X20	rs2891980	T/C	Intron	0.09	0.13	270,383
XRCC4	X21	rs1056503	T/G	Coding-synon	0.09	0.12	276,697

* Base change listed as Major allele / Minor allele

† Position obtained from the University of California, Santa Cruz Genome Browser ; Flanking = within 10 kb of either side of gene; Locus region = variation in region of gene, but not in transcript; mRNA-utr = variation in transcript, but not in coding region interval

‡ MAF = minor allele frequency using our panel of 94 breast cancer case and control subjects

§Applied Biosystems reported minor allele frequency in Caucasians

|| Corrected value. Applied Biosystems acknowledged error in reported minor allele frequency of 0.49 on their web site, but it has not been updated.

** NBS1 and RAD50 were not genotyped in the current study. All analyses for these two genes were performed using the raw genotype data freely available online from Applied Biosystems. Base change obtained from University of California, Santa Cruz Genome Browser.

**Table 2:** Haplotypes with frequency>0.01, LD group characterization and tSNPs selected using Utah genotyping data*

a. *ATM*																					
**A1**	**A2**	**A3**	**A4**	**A5**	**A6**	**A7**	**A8**	**A9**	**A10**	**A11**	**A12**	**A13**	**A14**	**Freq**							

1	1	1	1	1	1	1	1	1	1	1	1	1	1	0.54							
2	2	2	2	2	2	2	2	2	2	2	2	2	2	0.42							
2	2	2	2	2	2	1	2	2	2	2	2	2	2	0.01							
2	2	2	1	2	2	1	2	2	2	1	2	2	2	0.01							
1	1	1	1	1	1	1	1	1	1	2	1	1	1	0.01							
LD Group and tSNP Designation																					
1	1	1	1	1	1	1	1	1	1	1	1	1†	1								
b. *MRE11A*																					

**M1**	**M2**	**M3**	**M4**	**M5**	**M6**	**M7**	**M8**	**M9**	**M10**	**M11**	**Freq**										

2	2	2	1	2	2	1	1	1	1	1	0.30										
1	1	1	2	1	1	1	2	1	1	1	0.28										
1	1	1	1	1	1	1	1	1	1	1	0.25										
1	1	1	2	1	1	1	1	1	1	2	0.09										
1	1	1	1	1	1	1	2	1	1	1	0.06										
1	1	1	2	1	1	2	1	2	2	1	0.01										
LD Group and tSNP Designation																					
1	1	1	4†	1	1†	2	4	2	2†	3†											
c. *XRCC4*																					

**X1**	**X2**	**X3**	**X4**	**X5**	**X6**	**X7**	**X8**	**X9**	**X10**	**X11**	**X12**	**X13**	**X14**	**X15**	**X16**	**X17**	**X18**	**X19**	**X20**	**X21**	**Freq**

2	2	2	2	2	2	2	2	1	2	2	2	2	1	1	1	1	1	1	1	1	0.35
1	1	1	1	1	1	1	1	2	1	1	1	1	1	2	1	1	1	1	1	1	0.19
1	1	1	1	1	1	1	1	1	1	1	1	1	1	1	1	1	1	1	1	1	0.11
1	1	1	1	1	1	1	1	1	1	1	1	1	2	1	2	2	2	1	1	1	0.10
1	2	2	2	2	2	2	2	1	2	2	2	2	1	1	1	1	1	1	1	1	0.05
1	1	1	1	1	1	1	1	2	1	1	1	1	1	2	1	1	1	2	2	2	0.03
1	1	1	1	1	1	1	1	1	1	1	1	1	2	1	2	1	1	1	1	1	0.02
2	2	2	2	2	2	2	2	1	2	2	2	2	1	1	1	1	1	2	2	2	0.02
2	1	1	1	1	1	1	1	1	1	1	1	1	1	1	1	1	1	1	1	1	0.02
2	2	2	1	2	2	2	2	1	2	2	2	2	2	1	2	2	2	1	1	1	0.02
1	1	1	1	1	1	1	1	1	1	1	1	1	1	1	1	1	1	2	2	2	0.02
1	1	1	1	1	1	1	1	1	1	1	1	1	2	1	1	2	2	1	1	1	0.01
	1	1	1	1	1	1	2	1	1	1	1	1	2	1	1	2	2	1	1	1	0.01
LD Group and tSNP Designation																					
1	1†	1	1	1	1	1	1	4†	1	1	1	1	2†	4	2	2	2	3	3	3†	

* Analysis considers the total panel of 94 individuals together

† tSNP selected / group

**Table 3:** Comparison of LD groups for the Utah breast cancer cases and controls with Applied Biosystems (ABI) data*

**Gene**	**Group**	**Utah breast cancer case/control SNPs**	**Utah potential tSNPs**	**Utah % variance captured/group**	**ABI SNPs**	**ABI potential tSNPs**	**ABI % variance captured/group**
ATM	1	A1-A14	A1-A3, A5, A6, A8-A10, A12-A14	98.8%	A1-A14	A1-A3, A5, A8, A13, A14	98.2%
MRE11	1	M1, M2, M3, M5, M6	M1, M2, M3, M5, M6	100%	M1, M2, M3, M5, M6	M1, M2, M3, M5, M6	100%
	2	M7, M9, M10	M10	84.3%	M7, M9, M10	M7, M9, M10	100%
	3	M11	M11	100%	M11	M11	100%
	4	M4, M8	M4, M8	82.2%	M4, M8	M4, M8	83.9%
XRCC4	1	X1-X8, X10-X13	X2-X3, X5-X8, X10-X11	95.3%	X1-X8, X10-X13	X2, X3, X10, X11, X13	96.0%
	2	X14, X16-X18	X14	91.6%	X14, X16-X18	X14, X17, X18	93.5%
	3	X19-X21	X19-X21	100%	X19-X21	X19-X21	100%
	4	X9, X15	X9, X15	97.4%	X9, X15	X9, X15	96.8%

*We used Applied Biosystems' validated SNP genotype data for 45 Caucasian subjects.

**Table 4:** Haplotypes with frequency>0.01, LD group characterization and tSNP selected using data from Applied Biosystems*

a. *NBS1*										
**N1**		**N2**		**N3†**		**N4†**		**N5†**		**Frequency**

1		1		1		1		1		0.55
2		2		2		2		2		0.26
1		2		2		2		2		0.10
1		2		2		1		2		0.03
2		2		1		1		2		0.03
1		2		1		1		2		0.03
LD Group and tSNP Designation										
2‡		1‡		1		1		1		
b. *RAD50*										

**R1†**	**R2**	**R3†**	**R4†**	**R5**	**R6†**	**R7**	**R8**	**R9**	**R10**	**Frequency**

1	1	1	1	1	1	1	1	1	1	0.50
1	1	1	2	1	1	1	1	1	1	0.21
1	2	2	2	1	2	2	2	2	1	0.11
1	1	1	1	1	1	1	1	1	2	0.08
1	2	2	2	1	2	2	2	2	2	0.05
1	2	2	2	1	2	2	1	2	2	0.01
1	2	2	2	1	2	2	2	1	2	0.01
1	1	1	2	1	2	2	1	2	1	0.01
1	1	1	1	1	2	2	2	2	1	0.01
2	2	1	2	2	1	2	1	1	2	0.009§
LD Group and tSNP Designation										
2‡	1	1‡	1	2	1	1	1	1	3‡	

*We used Applied Biosystems' validated SNP genotype data for 45 Caucasian subjects.

† Allele designations have been changed from that listed by ABI to conform to the convention 1 = common allele, 2 = rare allele.

‡ tSNP selected / group

§ The haplotype with a frequency 0.009 was also analyzed to allow inclusion of rare variants at R1 and R5.
